# Pediatric Kidney Alone Versus Adult Simultaneous Kidney–Pancreas Recipients of Deceased Donor Kidneys: A Paired Analysis of 1675 Donors

**DOI:** 10.1111/petr.70506

**Published:** 2026-10-22

**Authors:** Joseph Sushil Rao, Shrihari Nagarajan, Prithvi Nagarajan, Ruining Zhou, Jando Danial, Ahalya Ramesh, Matthew Wright, Michael Chobanian, Michael F. Daily, Raja Kandaswamy, Abraham J. Matar

**Affiliations:** ^1^ Division of Transplantation, Department of Surgery University of Minnesota Minneapolis Minnesota USA; ^2^ Schulze Diabetes Institute, Department of Surgery University of Minnesota Minneapolis Minnesota USA; ^3^ School of Medicine University of Missouri‐Kansas City Kansas City Missouri USA; ^4^ Division of Transplantation, Department of Surgery Dartmouth Hitchcock Medical Center Hanover New Hampshire USA; ^5^ Division of Transplantation, Department of Surgery Albany Medical Center Albany New York USA

## Abstract

**Background:**

Allocation of high quality deceased donor kidneys to simultaneous pancreas–kidney (SPK) versus pediatric kidney alone (KI) recipients presents competing priorities regarding kidney allograft longevity and patient survival. We evaluated long‐term outcomes of contralateral kidneys transplanted into these distinct recipient populations to assess kidney allograft utility within this allocation framework.

**Methods:**

Using Scientific Registry of Transplant Recipients data from 2010–2025, we identified 1675 deceased donors whose kidneys were transplanted into an adult SPK recipient and a pediatric KI recipient. Outcomes included death censored graft survival (DCGS), patient survival, and freedom from composite graft failure or death. Kaplan–Meier analyses were performed with survival estimates at 1, 3, 5, and 10 years and log‐rank comparisons.

**Results:**

Ten‐year DCGS was significantly higher among adult SPK recipients than among pediatric KI recipients (82.1% versus 70.6%; *p* = 0.0001). Conversely, 10‐year patient survival was substantially higher among pediatric KI recipients (95.4% versus 78.3%; *p* = 0.0001). Despite these differences, 10‐year freedom from composite graft failure or death was comparable between SPK and pediatric KI recipients (69.1% versus 67.9%; *p* = 0.47).

**Conclusions:**

Among contralateral kidneys from the same deceased donors, adult SPK recipients demonstrated superior DCGS, whereas pediatric KI recipients experienced substantially better patient survival. Comparable long‐term composite outcomes highlight the competing considerations of graft longevity and recipient survival in deceased donor kidney allocation. These findings suggest that assessments of kidney allograft utility should consider both graft‐specific and recipient‐centered outcomes rather than relying on either endpoint independently.

## 1 Introduction

Allocation of high quality deceased donor kidneys to simultaneous pancreas‐kidney (SPK) candidates remains controversial when those same kidneys might instead be used for pediatric kidney‐alone (KI) recipients [1]. More broadly, kidney allocation policy often requires balancing competing definitions of transplant utility, including graft longevity, patient survival, and the total benefit derived from scarce deceased donor organs [1]. This tension is particularly relevant when comparing adult SPK and pediatric KI recipients because both groups may receive priority for high quality kidneys [2], but for different clinical and policy reasons.

Prior multiorgan paired kidney analyses in simultaneous liver‐kidney and simultaneous heart‐kidney transplantation have raised concern that multiorgan allocation may not always maximize kidney allograft utility [3], but analogous data for the specific tradeoff between adult SPK and pediatric KI recipients are limited. A paired kidney comparison offers a useful framework for examining this question because kidneys from the same donor can be evaluated across two recipient populations with markedly different biology, comorbidity burden, and long‐term risk profiles [2, 4]. We therefore examined donor matched kidneys in which one kidney was transplanted as part of an adult SPK and the contralateral kidney was transplanted into a pediatric KI recipient to evaluate graft utility under this allocation scenario.

## 2 Methods

This study used data from the Scientific Registry of Transplant Recipients (SRTR). The SRTR data system includes data on all donor, wait‐listed candidates, and transplant recipients in the United States, submitted by the members of the Organ Procurement and Transplantation Network (OPTN). The Health Resources and Services Administration (HRSA), U.S. Department of Health and Human Services provides oversight to the activities of the OPTN and SRTR contractors. Using SRTR data, we identified 1675 deceased donor kidney pairs transplanted between 2010 and 2025 in which one kidney was allocated to an adult SPK recipient and the contralateral kidney to a pediatric KI recipient. Outcomes included DCGF, patient survival (PS), and composite graft failure/death (CGF). Kaplan–Meier analyses with landmark comparisons at 1, 3, 5, and 10 years were performed.

By restricting the analysis to contralateral kidneys from the same deceased donor, this design directly controls for donor‐level characteristics that commonly confound conventional comparisons between pediatric KI and adult SPK recipients. This approach therefore permits a more focused assessment of how recipient phenotype relates to long‐term graft and patient outcomes within a real‐world allocation setting.

## 3 Results

Among these 1675 donor pairs, death censored kidney graft survival was higher for SPK recipients than for pediatric KI recipients, with 1‐, 3‐, 5‐, and 10‐year graft survival of 99.0%, 95.6%, 91.0%, and 82.1% versus 98.9%, 93.4%, 86.3%, and 70.6%, respectively (log‐rank *p* = 0.0001) (Figure 1A). In contrast, overall patient survival was substantially higher among pediatric KI recipients; 1‐, 3‐, 5‐, and 10‐year patient survival was 98.3%, 95.9%, 92.9%, and 78.3% in SPK recipients versus 99.6%, 99.0%, 98.3%, and 95.4% in pediatric KI recipients (log‐rank *p* = 0.0001) (Figure 1B). Long‐term freedom from the composite outcome of death or graft failure was similar between groups, with 1‐, 3‐, 5‐, and 10‐year rates of 97.4%, 92.2%, 85.7%, and 69.1% for SPK recipients versus 98.4%, 92.5%, 85.0%, and 67.9% for pediatric KI recipients (log‐rank *p* = 0.47) (Figure 1C).

**FIGURE 1:**
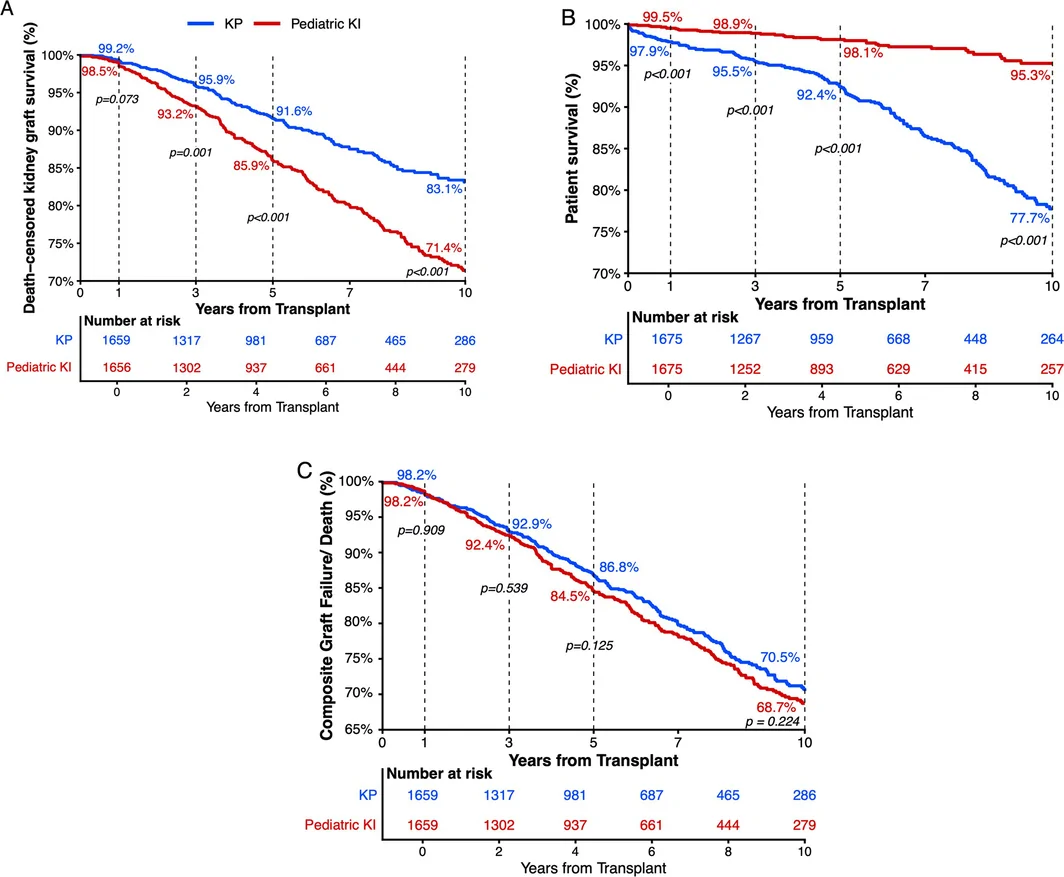
Paired kidney outcomes comparing adult simultaneous kidney‐pancreas transplantation recipients and pediatric kidney alone recipients for (A) death‐censored graft survival, (B) patient survival, (C) composite graft failure/death. Survival estimates were calculated using Kaplan‐Meir methodology. Pairwise landmark comparisons at 1, 3, 5, and 10 years were performed using a log‐rank test. Number at risk is displayed below each panel.

## 4 Discussion

The comparison between pediatric KI and adult SPK recipients brings a longstanding allocation dilemma into sharper focus [5]. Among kidneys from the same deceased donors, SPK recipients achieved superior death censored kidney allograft survival, whereas pediatric recipients derived markedly better overall patient survival. These findings suggest that allocation in this setting does not favor a single dominant endpoint but instead reflects competing priorities depending on whether the emphasis is placed on graft longevity or recipient survival.

This divergence likely reflects important differences in recipient biology and clinical context rather than donor quality, because the paired kidney design substantially minimizes donor related confounding. Inferior graft survival among pediatric recipients may relate to developmental, psychosocial, adherence, and transition of care challenges that have historically complicated long‐term pediatric and adolescent transplant outcomes [5]. Conversely, higher mortality among SPK recipients despite functioning kidney allografts likely reflects the cumulative comorbidity burden and systemic consequences of long‐standing diabetes.

The similarity in long‐term composite outcomes is particularly important from a policy standpoint. These findings suggest that allocation to adult SPK recipients may better preserve kidney graft longevity, whereas allocation to pediatric recipients may better maximize patient survival benefit. Framing the question in this way may be more useful than asking whether one recipient group simply represents a better use of the kidney, because the answer depends on which outcome the allocation system seeks to prioritize.

Several limitations merit consideration. As a retrospective registry analysis, this study cannot fully account for unmeasured differences in comorbidity burden, adherence, center practices, immunosuppression, transition readiness, or social determinants of health that may differentially influence outcomes in pediatric and adult recipients. In addition, registry data cannot fully define the mechanisms underlying graft loss or the pathways through which pediatric developmental factors and SPK related metabolic factors shape long‐term transplant outcomes. Nevertheless, the paired kidney design provides a uniquely informative framework for evaluating the tradeoffs inherent in allocating high quality deceased donor kidneys between adult SPK and pediatric KI candidates.

## Data Availability

The data that support the findings of this study are available from The SRTR Database. Restrictions apply to the availability of these data, which were used under license for this study. Data are available from https://www.srtr.org/transplant‐professionals/reports/the‐srtr‐database/ with the permission of The SRTR Database.
